# The psychological impact of COVID-19 pandemic on healthcare workers

**DOI:** 10.3389/fpubh.2022.963673

**Published:** 2022-08-17

**Authors:** Fei Tong, Lemeng Zhang, Liping Huang, Hongxia Yang, Minni Wen, Ling Jiang, Ran Zou, Feng Liu, Wanglian Peng, Xufen Huang, Desong Yang, Hui Yang, Lili Yi, Xiaohong Liu

**Affiliations:** ^1^Department of Clinical Spiritual Care, Hunan Cancer Hospital/The Affiliated Cancer Hospital of Xiangya School of Medicine, Central South University, Changsha, China; ^2^Medical Services Section, Xinhua People's Hospital, Pingdingshan, China; ^3^Medical Oncology Department, Xinhua People's Hospital, Pingdingshan, China

**Keywords:** COVID-19, anxiety, depression, healthcare workers, post-traumatic stress disorder (PSTD), fear

## Abstract

**Background:**

As unprecedented and prolonged crisis, healthcare workers (HCWs) are at high risk of developing psychological disorders. We investigated the psychological impact of COVID-19 pandemic on HCWs.

**Methods:**

This cross-sectional study randomly recruited 439 HCWs in Hunan Cancer Hospital via a web-based sampling method from June 1st 2021 to March 31st 2022. Anxiety and depression levels were measured using Hospital Anxiety and Depression Scale (HADS). The Post Traumatic Stress Disorder (PTSD) Checklist for DSM-5 (PCL-5) was used to assess the presence and severity of PTSD. Fear was measured by modified scale of SARS. Data were collected based on these questionnaires. Differences in fear, anxiety, depression and PTSD among HCWs with different clinical characteristics were analyzed using a multivariate analysis of variance. The Cronbach's alpha scores in our samples were calculated to evaluate the internal consistency of HADS, fear scale and PCL-5.

**Results:**

The prevalence of anxiety, depression, and PTSD in HCWs was 15.7, 9.6, and 12.8%, respectively. Females and nurses were with higher fear level (*P* < 0.05) and higher PTSD levels (*P* < 0.05). Further analysis of female HCWs revealed that PTSD levels in the 35–59 years-old age group were higher than that in other groups; while married female HCWs were with increased fear than single HCWs. The internal consistency was good, with Cronbach's α = 0.88, 0.80 and 0.84 for HADS, fear scale, and PCL, respectively.

**Conclusion:**

Gender, marital status, and age are related to different level of psychological disorders in HCWs. Clinical supportive care should be implemented for specific group of HCWs.

## Introduction

The coronavirus disease 2019 (COVID-19) is a serious threat to global public health and economic stability (1). First emerged in December 2019 (2) and with rapid spread worldwide, COVID-19 was declared as a global pandemic (3). As an unprecedented and prolonged long-lasting crisis, healthcare workers (HCWs) on the front line are at high risk of developing psychological disorders (4).

The psychological impact of COVID-19 included fear, anxiety, depression, burnout, and fatigue (5). Owing to the risk of virus exposure, high workload demand, and distressing work shifts, HCWs are at high risk to develop psychological disorders (6). An umbrella review of systematic reviews and meta-analyses have revealed that the COVID-19 pandemic exerts profound impacts on the mental health states of HCWs and leads to high levels of anxiety, depression, post-traumatic stress disorder (PTSD), sleep disorders, and burnout (7). A another meta-analysis reported that among 159,194 healthcare providers in Asia, the pooled prevalence of depression, anxiety, stress, fear, and burnout was 37.5, 39.7, 36.4, 71.3, and 68.3%, respectively. The risk of developing depression and anxiety was increased in females and nurses (8). In Italy, the prevalence of depression, anxiety, and insomnia among HCWs was 50.4, 44.6, and 34.0%, respectively, and most participants were female, nurses, married, and working in tertiary hospitals (9). Symptoms of depression (50.4%), insomnia (34.0%), anxiety (44.6%), and distress (71.5%) have also been reported among HCWs in China (10). A high prevalence of PTSD (20.8%) among HCWs was reported at Central Hospital of Wuhan after the first outbreak of COVID-19 (11). These psychological disorders caused by the COVID-19 pandemic can result in adverse consequences, such as suicide and high turnover intention. The high turnover intention leads to staffing shortages and increases pressure on HCWs, which may increase burnout, adverse incidents, and patient harm, leading to future problems for the resilience of healthcare system (12). Given the ongoing and unpredictable pandemic, protecting HCWs against psychological disorders is crucial to maintain the availability of healthcare services (13). Focusing on and minimizing the psychological impacts of this pandemic on HCWs remain a challenge for healthcare systems worldwide.

HCWs usually differ in their perceived levels of anxiety and depression due to various factors, including gender, marital status, specialty and service provided, and duration of employment (14). It has been reported that younger age, longer quarantine, and lack of practical support were risk factors for psychological distress (15). The fear of being infected, struggling with difficult emotions, witnessing hasty end-of-life decisions, and inability to care for family were modifiable determinants of symptoms of psychological disorders (16). Therefore, the exploration of risk factors for psychological disorders is important.

As a susceptible population, cancer patients are often accompanied by clue symptoms such as fever, cough and dyspnea. Additionally, delayed treatment and hypochondriac tendency are more likely to cause psychological crisis in cancer patients, which may also affect HCWs in cancer center. There are a large number of researches on the psychological disorders in HCWs in general hospital, however, few study focus on the psychological disorders of HCWs and their related factors in cancer center during the COVID-19 pandemic. To this end, we evaluated the prevalence of psychological disorders among HCWs in Hunan Cancer Hospital and investigated the potential risk factors contributing to psychological disorders. Our findings provide important evidences to guide the promotion of psychological health among HCWs.

## Methods

### Study design and participants

This cross-sectional, survey-based study was carried out to evaluate the impact of COVID-19 on psychological disorders among HCWs. HCWs, including physicians, surgeons, and nurses, were randomly recruited from existing personal and professional contacts via a web-based sampling method from June 1st 2021 to March 31st 2022. Participants provided consent by virtually agreeing to participate as part of the sampling process. Owing to pandemic prevention and control measures, the questionnaire was completed online. A screening form was used to ensure that participants met our inclusion criteria, and an electronic informed consent was included at the beginning of the online questionnaire. The consent form included information about the purpose of the study as well as the principal investigator's contact information. At the end of the online questionnaire, participants were invited to share the link with others who met the inclusion criteria. Participants with mental illness, or hearing, speech, or cognitive impairment that may prevent them from completing the questionnaire on their own were excluded. This study was approved by the ethics committee of Hunan Cancer Hospital.

### Data collection

Participants' clinical information, including age, gender, marital status, duration of employment, and service provided were collected based on the online questionnaire. The questionnaire was prepared by the qualitative research team and was analyzed by each investigator. This study was conducted by physicians, psychologists and medical staff who had received systematic scientific research training.

### Psychological outcomes

#### Anxiety and depression assessment

Anxiety and depression levels were determined using the Chinese 14-item Hospital Anxiety and Depression Scale (HADS) (17). The questions in these subscales asked participants to recall their mental presentation under each scenario by answering always, usually, sometimes, rarely, or never. Subscales were assessed separately, with cumulative scores of 0–7, 8–10, 11 or higher indicating normal, borderline abnormal (borderline case), and abnormal, respectively.

#### Fear measurement

An 18-item scale was used to measure fear related to COVID-19. The scale was based on a previous scale used to assess fear among HCWs during the SARS (18). The items included fear of becoming infected, fear of caring for COVID-19 patients, fear of infecting others, and fear of death. Participants responded to the 18 items on a 4-point Likert scale, with 0, 1, 2, and 3 indicating definitely false, somewhat false, somewhat true, and definitely true, respectively.

#### Evaluation of PTSD

A total of 20 items are included, which correspond to the diagnostic criteria set by the Diagnostic and Statistical Manual of Mental Disorders (DSM-5). The PTSD Checklist for DSM-5 (PCL-5) was used to assess the presence and severity (19). Participants reported their symptoms based on the 20 items using a 5-point intensity scale ranging from 0 (nothing) to 4 (extremely). A higher cumulative score for each item indicates a higher degree of PTSD symptoms.

### Statistical analysis

All statistical analyses were performed using Stata version 13.1 (Stata Corp., College Station, TX, USA). Fear, anxiety, depression and PTSD levels were expressed as mean ± standard deviation (*SD*). Differences in the aforementioned levels between HCWs with different clinical characteristics were analyzed using a multivariate analysis of variance (MANOVA). The Cronbach's alpha scores in our samples were calculated to evaluate the internal consistency of HADS, fear scale, and PCL-5. A two-tailed *P* < 0.05 was considered statistically significant.

## Results

### Demographic characteristics of the participants

A total of 439 HCWs were included in this study. The demographic characteristics of the participants are reported in Table 1. Total 439 HCWs were enrolled. Among them, 280 (63.9%) were nurses, 83 (36.1%) were clinicians. Most participants were female (360 [82.0%]) and were married (293 [66.7%]). 184 (41.9%) were <35 years-old; 215 (49.0%) were aged 35 to 59. Duration of employment and service provided and other demographic Characteristics of participants were also listed in Table 1.

**Table 1:** Demographic characteristics of participants.

**Characteristics**	**No. (%)**
**Gender**	
Female	360 (82.0%)
Male	79 (18.0%)
**Age (years-old)**	
≤35	184 (41.9%)
35–59	215 (49.0%)
≥60	40 (9.1%)
**Marital status**	
Married	293 (66.7%)
Single	146 (33.3%)
**Occupation**	
Nurses	280 (63.9%)
Clinicians	83 (36.1%)
**Duration of employment**	
<5 years	135 (30.8%)
6–10 years	140 (31.9%)
11–20 years	95 (21.6%)
More than 20 years	69 (15.7%)
**Service provided**	
Outpatient	51 (11.6%)
Inpatient	295 (67.2%)
Inpatient and outpatient	93 (21.2%)

### Reliability

The internal consistency (Cronbach's α) of was good, with α = 0.88 for HADS, α = 0.80 for fear scale, and α = 0.84 for PCL.

### The prevalence of psychological disorders of HCWs

Based on the questionnaire survey, the prevalence of anxiety, depression, and PTSD in the HCWs were 15.7, 9.6, and 12.8%, respectively. The prevalence of borderline abnormal anxiety and depression were 27.1 and 21.0%. The prevalence of anxiety, depression, and PTSD has been listed in Table 2.

**Table 2:** The prevalence of anxiety, depression, and PTSD.

	**Prevalence (%)**
**Anxiety level**	
Abnormal	15.7
Borderline abnormal (borderline)	27.1
Normal	56.9
**Depression level**	
Abnormal	9.6
Borderline abnormal (borderline)	21.0
Normal	69.4
**PTSD level**	
Abnormal	12.8
Normal	87.2

### Association clinical characteristics between fear, anxiety, depression, PTSD

The risk factors for fear, anxiety, depression, PTSD, and hope levels were investigated. The results revealed that fear level was significantly associated with gender (*P* = 0.002) and occupation (*P* = 0.013); while PTSD levels were significantly associated with gender (*P* = 0.012) and occupation (*P* = 0.035). Females and nurses were more inclined to develop fear and PTSD. Among HCWs, females and nurses were risk factors for fear and PTSD. While Age, marital status, duration of employment, and service provided were not related to psychological disorders. Association between fear, anxiety, depression, PTSD and clinical characteristics of HCWs has been listed in Table 3.

**Table 3:** Association between fear, anxiety, depression, PTSD and clinical characteristics of HCWs.

	**Fear**		**Anxiety**		**Depression**		**PTSD**	
	**Mean *±SD***	***P***	**Mean *±SD***	***P***	**Mean *±SD***	***P***	**Mean *±SD***	***P***
**Gender**		0.002		0.569		0.936		0.012
Male	21.08 ± 10.40		6.61 ± 3.50		5.59 ± 3.81		12.32 ± 9.62	
Female	25.65 ± 10.90		6.89 ± 3.66		5.55 ± 3.50		16.54 ± 12.81	
**Age (years-old)**		0.473		0.125		0.431		0.062
≤35	24.43 ± 11.37		6.52 ± 3.49		5.40 ± 3.37		14.50 ± 11.66	
35–59	25.23 ± 10.60		7.09 ± 3.73		5.68 ± 3.69		16.88 ± 12.92	
≥60	23.73 ± 11.02		6.59 ± 3.42		5.55 ± 3.43		13.87 ± 10.98	
**Marital status**		0.106		0.073		0.923		0.796
Married	25.44 ± 10.62		7.05 ± 3.70		5.57 ± 3.62		15.93 ± 12.51	
Single	23.46 ± 11.64		6.32 ± 3.41		5.53 ± 3.38		15.57 ± 12.25	
**Occupation**		0.013		0.527		0.824		0.035
Nurse	25.81 ± 10.72		6.92 ± 3.60		5.53 ± 3.40		16.66 ± 13.05	
Clinician	22.86 ± 11.19		6.67 ± 3.72		5.62 ± 3.85		14.01 ± 10.75	
**Duration of employment**		0.632		0.465		0.734		0.098
≤5 years	24.32 ± 10.24		6.70 ± 3.49		5.33 ± 3.49		14.26 ± 10.99	
6–10years	23.96 ± 12.07		6.86 ± 3.21		5.66 ± 3.64		15.54 ± 11.17	
11–20years	24.15 ± 12.21		7.01 ± 3.48		5.54 ± 3.36		15.88 ± 10.95	
≥20 years	23.78 ± 11.54		6.88 ± 3.72		5.68 ± 3.79		16.01 ± 11.76	
**Service provided**		0.526		0.523		0.428		0.268
Outpatient	24.11 ± 10.98		6.84 ± 3.41		5.71 ± 3.76		14.58 ± 10.54	
Inpatient	23.54 ± 11.47		6.52 ± 3.43		5.69 ± 3.88		14.88 ± 10.42	
Inpatient and outpatient	22.86 ± 11.19		6.98 ± 3.71		5.65 ± 3.81		14.98 ± 10.66	

### Association between fear, anxiety, depression, PTSD in female HCWs

We further analyzed the risk factors for the aforementioned levels in female HCWs. The results revealed that the fear levels in married HCWs were significantly higher than those in single HCWs (*P* = 0.039). And the PTSD levels of HCWs in the 35–59 age group were remarkably higher than in other groups (*P* = 0.042). Among female HCWs, married status was a risk factor for fear, and age was a risk factor for PTSD. Association between fear, anxiety, depression, PTSD and clinical characteristics of female HCWs has been listed in Table 4.

**Table 4:** Association between fear, anxiety, depression, PTSD, and clinical characteristics of female HCWs.

**Characteristics**	**Number**	**Fear**		**Anxiety**		**Depression**		**PTSD**	
		**Mean *±SD***	***P***	**Mean *±SD***	***P***	**Mean *±SD***	***P***	**Mean *±SD***	***P***
Age (years-old)			0.459		0.105		0.391		0.042
≤35	154	25.18 ± 11.28		6.54 ± 3.42		5.38 ± 3.38		14.24 ± 11.79	
35–59	170	26.08 ± 10.55		7.20 ± 3.86		5.78 ± 3.54		17.87 ± 13.57	
≥60	36	25.84 ± 11.85		6.97 ± 3.68		5.55 ± 3.45		14.83 ± 11.02	
Marital Status			0.039		0.086		0.638		0.532
Married	252	27.02 ± 10.46		6.34 ± 3.39		5.61 ± 3.57		16.83 ± 13.02	
Single	108	23.14 ± 11.70		7.12 ± 3.76		5.41 ± 3.32		15.86 ± 12.36	
Occupation			0.375		0.566		0.992		0.341
Nurse	291	25.92 ± 10.77		6.94 ± 3.59		5.55 ± 3.39		16.87 ± 12.03	
Clinician	69	24.55 ± 11.49		6.65 ± 3.98		5.55 ± 3.95		15.15 ± 11.86	
Duration of employment			0.562		0.352		0.452		0.442
≤5 years	96	24.55 ± 11.49		6.12 ± 3.45		5.62 ± 3.77		16.01 ± 12.98	
6–10years	104	24.55 ± 11.49		5.98 ± 3.66		5.35 ± 3.18		16.54 ± 12.66	
11–20years	75	24.55 ± 11.49		6.52 ± 3.18		5.58 ± 3.44		16.99 ± 13.15	
≥20 years	85	24.55 ± 11.49		6.01 ± 3.55		5.52 ± 3.49		16.78 ± 12.98	
Service provided			0.482		0.158		0.658		0.754
Outpatient	41	24.55 ± 11.49		6.05 ± 3.44		5.49 ± 3.33		16.55 ± 12.98	
Inpatient	244	24.55 ± 11.49		6.61 ± 3.87		5.79 ± 3.15		16.66 ± 13.14	
Inpatient and outpatient	75	24.55 ± 11.49		6.48 ± 3.12		5.54 ± 3.17		16.97 ± 13.55	

## Discussion

The high infection risk, rapid spread, and prolonged uncertainty of COVID-19 have aggravated the physical and mental burden on all HCWs (20). Our data present concerns regarding the psychological disorders of HCWs during the COVID-19 pandemic.

During the pandemic, HCWs have been at the forefront of the global fight against the virus; therefore, they face unprecedented scenarios, often beyond their ordinary levels of experience and training. Moreover, HCWs are in direct contact with the patient and more exposed to high stress environment and workplace violence, which increases psychological stresses and occupational burnout of HCWs (21, 22). Owing to prolonged uncertainty, social isolation, and evolving professional demands, the pandemic poses unprecedented threats. Therefore, this pandemic can be regarded as a disaster in which the number of victims and medical needs exceed the capabilities and capacities of the existing healthcare system (23). HCWs constitute the most important groups who are involved in caring disaster victims, suggesting they should be involved in all phases of disaster risk management, such as risk assessment, pre-disaster planning, response during crises, and risk mitigation throughout reconstruction.

It has been reported that clinicians are at a high risk of long-term psychological disorders during the pandemic (24, 25). Another meta-analysis of 38 studies with 53,784 HCWs indicated that the pooled prevalence of mental health problems such as PTSD, anxiety, depression, and distress in HCWs during the pandemic was 49, 40, 37, and 37%, respectively (26). Sahebi et al. conducted an umbrella review and meta-analysis and demonstrated that there is a high prevalence of PTSD (13.52%) among HCWs during the pandemic (27). In this cross-sectional study, we investigated the psychological disorders of HCWs. Overall, 15.7, 9.6, and 12.8% of HCWs reported symptoms of anxiety, depression, and PTSD, respectively. The prevalence of psychological disorders is relatively low compared with other studies. The potential explanation includes the control of COVID-19 in China, the emphasis of mental health and social support. And another important aspect is that clinical spiritual care has been routinely used for HCWs in our study. Identification of spiritual needs, understanding the specific needs, developing the individual spiritual care plan, which might contribute to relatively lower prevalence of psychological disorders (28). While release negative emotions, avoid overwork, maintain proper physical exercise, take advantage of social support system, might be useful to maintain psychological health (29). To better approach the practical and daily dimensions of spiritual care and to better address and consider the individual specific spiritual needs might be special important during COVID-19 pandemic.

HCWs may be at a higher risk of psychological disorders than the general population. Within the healthcare workforce, nurses and clinicians usually differ in their perceived levels of anxiety and depression due to various factors including gender, marital status, specialty and service provided, and duration of employment (30). Nurses are prone to varying degrees of psychological disorders during the pandemic (31, 32). A previous meta-analysis of 30 studies with a combined total of 33,062 HCWs revealed that females and nurses were with higher rates of affective symptoms than males during the pandemic (33). Another study also reported a high prevalence of symptoms of anxiety, depression, and PTSD in HCWs while nurses were with the highest prevalence (16). Accumulating studies have demonstrated that anxiety, depression, and stress were prevalent among nurses, and it was essential to develop psychological interventions that could improve the mental health of nurses during the pandemic (34, 35). Consistent with these findings, we also found that females and nurses were risk factors for fear and PTSD in HCWs during the pandemic. Moreover, we found that among female HCWs, married status was a risk factor for fear and age was a risk factor for PTSD. Being more afraid of infecting their families or unable to care for their families may partially explain the psychological burdens of married and female HCWs. Thus, there is a need for occupational health surveillance and workplace health promotions programs, especially in the long-term for prevention, early diagnosis and promotion of mental health of HCWs (36).

For disaster risk management, more interventions or appropriate social supports should be implemented to specific groups. For instance, the COVID-19 pandemic may be related the exacerbation of psychological problems especially depression in high-risk population such as pregnant women and the postpartum period (37). Moreover, human resource supply is one of the most vital factors in the production and delivery of services (38). Staffing shortages threaten healthcare systems, which may be unable to meet the current and next global challenges and can further increase HCWs' workloads and lead to burnout (12). Given the essential role of HCWs, especially during a pandemic, interventions such as efforts to recruit new staff, reduce workloads, offer financial support are required. Also, to help HCWs especially nurses to tackle and endure burnout in the pandemic, it is recommended to include the promotion of resilience in the design of interventions to reduce burnout as resilience is a protective criterion for home burnout (39). HCWs should maintain proper physical exercise, release negative emotions, and seek psychological counseling if necessary. Furthermore, there is a positive correlation between the organizational commitment and the quality of working life of HCWs (40). More emphasis should be placed on the optimization of work environment to strengthen the commitment to the organization, making training programs to increase disaster response expertise, and establishment of a fair payment system to improve working enthusiasm of HCWs. The present study has several limitations. First, psychological outcomes evaluation was based on online questionnaire. In future studies, the use of clinical interviews for comprehensive evaluation of the problem is encouraged. Second, this study did not confirm prospectively the function of clinical spiritual care in HCWs. Futher study should be designed to explore the function of clinical spiritual care. Lastly, this study adopted a cross-sectional design. A longitudinal study is required to evaluate the prevalence of psychological burden with the progression of COVID-19.

## Conclusion

In conclusion, gender, marital status and age are related to different level of psychological disorders in HCWs. Female and nurse HCWs are prone to developing psychological disorders. Clinical supportive care should be implemented to improve the psychological health levels for specific group of HCWs during the unpredictable long lasting pandemic.

## Data availability statement

The raw data supporting the conclusions of this article will be made available by the authors, without undue reservation.

## Ethics statement

The studies involving human participants were reviewed and approved by Hunan cancer hospital. Written informed consent to participate in this study was provided by the participants' legal guardian/next of kin.

## Author contributions

FT, LZ, and XL carried out the conception and design of the research. LH, HoY, RZ, WP, and HuY participated in the acquisition of data. XH, LY, MW, LJ, and FL carried out the analysis and interpretation of data. XL participated in the design of the study, prepare and revise the manuscript. All authors read and approved the final manuscript.

## Funding

This study was supported by grants from Natural Science Foundation of Changsha Science and technology Bureau (kp2001024), Clinical medical technology innovation guided project (2020SK51112) and National Natural Science Foundation of Hunan Province (2020RC3067).

## Conflict of interest

The authors declare that the research was conducted in the absence of any commercial or financial relationships that could be construed as a potential conflict of interest.

## Publisher's note

All claims expressed in this article are solely those of the authors and do not necessarily represent those of their affiliated organizations, or those of the publisher, the editors and the reviewers. Any product that may be evaluated in this article, or claim that may be made by its manufacturer, is not guaranteed or endorsed by the publisher.
